# Architected fibrous scaffolds for engineering anisotropic tissues

**DOI:** 10.1088/1758-5090/ac0fc9

**Published:** 2021-07-27

**Authors:** James Alexander Reid, Kiera D Dwyer, Phillip R Schmitt, Arvin H Soepriatna, Kareen LK Coulombe, Anthony Callanan

**Affiliations:** 1 Institure for Bioengineering, School of Engineering, The University of Edinburgh, Edinburgh, United Kingdom; 2 Center for Biomedical Engineering, Brown University, Providence, RI 02912, United States of America; 3 Joint first authorship.

**Keywords:** electrospinning, tissue engineering, scaffold, fiber, anisotropy, biomaterial, regenerative medicine

## Abstract

Mimicking the native three-dimensional microenvironment is of crucial importance when biofabricating a new healthcare material. One aspect of the native tissue that is often omitted when designing a suitable scaffold is its anisotropy. Not only is matching native mechanical properties important when designing implantable scaffolds or healthcare materials, but matching physiological structure is also important as many cell populations respond differently to fiber orientation. Therefore, novel aligned electrospun scaffolds with varying fiber angles and spacing of bundles were created and mechanically characterized. Through controlling the angle between the fibers in each layer of the scaffold, a range of different physiological anisotropic mechanical properties were achieved that encompasses values found in native tissues. Extrapolation of this mechanical data allowed for any native tissue’s anisotropic Young’s modulus to be mimicked by electrospinning fibers at a particular angle. These electrospun scaffolds were then incorporated with cell-laden hydrogels to create hybrid structures that contain the benefits of both scaffolding techniques with the ability to encapsulate cells in the hydrogel. To conclude, this study develops a novel bundled fiber scaffold that was architected to yield anisotropic properties matching native tissues.

## 1. Introduction

Tissue engineering is a multidisciplinary field that combines concepts from engineering and life sciences in an effort to develop healthcare materials that can be applied to regenerative medicine [1]. In recent years there has been a particular interest in mimicking the native three-dimensional (3D) tissue microenvironment, both in terms of physical structure and biological makeup [2].

Most tissues have a degree of anisotropy in both their physiological and mechanical properties [3, 4]. Being able to mimic these anisotropic properties is of paramount importance in the field of tissue engineering as these properties drive cell adhesion, differentiation and proliferation [5]. Tissue anisotropy with regards to bulk mechanical properties can range from close to none (isotropy) in tissues such as the liver, up to a high degree of anisotropy in tissues such as ligaments and tendons [3, 6]. Physiological anisotropy can also be found at smaller length scales in the orientation of extracellular matrix fibers. For example, myocardial fiber orientation varies continuously through the myocardial wall and is important for both mechanical and electrical myocardial function [4]. Likewise, tissues such as ligaments and tendons have highly anisotropic fiber orientations to stabilize articulating joints and transmit forces from the muscles to the bones to enable skeletal movement [7].

Electrospinning is a manufacturing method widely used with different polymers such as polycaprolactone (PCL) to generate fibrous materials. Its versatility allows for a range of different fiber sizes and orientations to be generated using various polymers and solvents [8]. For this reason, electrospinning has been used in all facets of tissue engineering, with applications ranging from wound healing to bypass grafts [9–18]. Mimicking anisotropy using electrospun fibers has been widely reported in the literature, albeit the majority of these materials have used either fully aligned fibers or randomly orientated fibers [19–23]. For example, increasing mandrel rotational speed (0–3000 RPM) increased the alignment of electrospun fibers and led to an upregulation of myosin heavy chain *α* (MYH6) in seeded cardiomyocytes [24]. Fiber orientation and size were both shown to influence endothelial cell (EC) phenotype, with the highly anisotropic fibers leading to increased proliferation and cell alignment [25]. Likewise, work examining mesenchymal stem cell motility and migration on aligned and random fibers found that aligned fibers enhanced migration velocity and increased expression of key phenotypic genes [20]. As expected, they discovered that PCL is a commonly used polymer for healthcare materials and in the field of tissue engineering due to its versatility in terms of scaffold manufacturing and its biocompatible and biodegradable properties [26–28]. Many studies have shown that PCL can be used in conjunction with electrospinning to generate scaffolds with physiologically relevant mechanical properties [27, 29]. Furthermore, it has been shown that the degradation rate of PCL can be accurately controlled and that its degradation by-products have negligible negative effects on mouse bone marrow mesenchymal stem cells *in vitro* [30, 31]. Moreover, PCL has been successfully used in a variety of different implants covering a wide range of applications [32].

The present study develops and quantifies a controlled system which enables a range of electrospun PCL scaffolds with tuneable physiologically relevant anisotropic biomechanical properties to be manufactured and incorporated into cell laden hydrogels. Novel aligned electrospun scaffolds with carefully varied fiber angles and bundled fibers were created and mechanically characterized. Electrospun scaffolds were specifically designed to contain large gaps between each bundle of fibers to facilitate cell and hydrogel infiltration. A constitutive model of the mechanical data allows for any native tissue’s anisotropic ratio of longitudinal to transverse Young’s moduli (referred to as anisotropic ratio going forward) to be recreated by electrospinning fibers at a defined angle. We combined these scaffolds with cell-laden hydrogels composed of neonatal human dermal fibroblasts (NHDFs) to create viable, composite engineered tissues, whose mechanical and structural properties could be influenced by the architectured electrospun PCL scaffolds. Further, we successfully co-cultured the NHDFs with ECs to illustrate the system’s tunability and diversity in designing hybrid scaffolds for applications in tissue engineering and regenerative medicine.

## 2. Experimental section

### 2.1. Electrospinning

PCL (Mn = 80 000, 20% w/v, Sigma Aldrich) was added to hexafluoroisopropanol (15 ml, Manchester Organics) and dissolved overnight on a tube roller. Solutions were placed into a 20 ml syringe and pumped using an EP-H11 syringe pump (Harvard Apparatus) into an EC-DIG electrospinner (IME Technologies).

**Figure 1.**
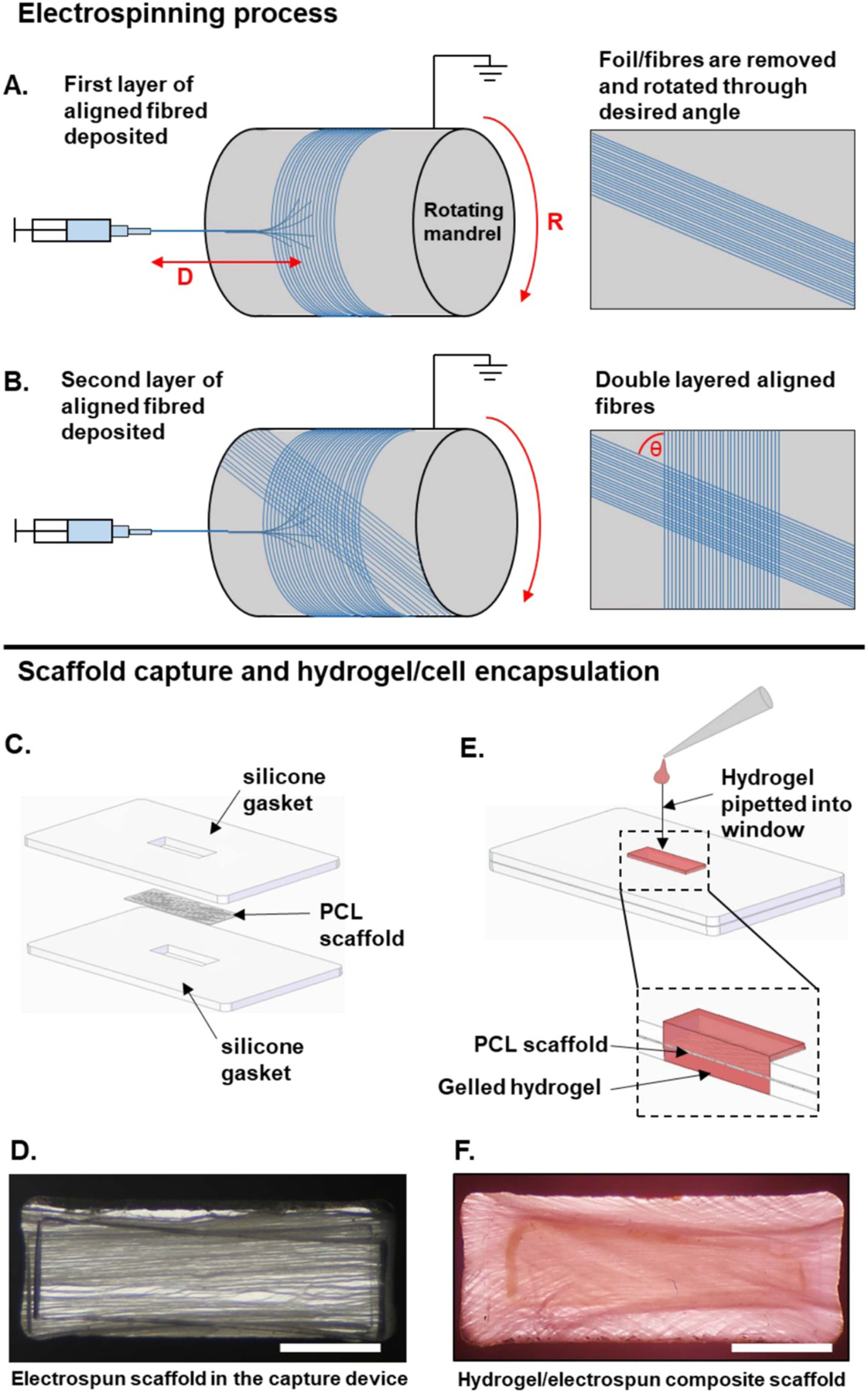
Diagrammatic representation of the architected scaffolds’ manufacturing process. (A) and (B) represent the scaffold manufacturing method. (C)–(F) represent the intended use of the electrospun scaffold when encapsulated in a hydrogel. (A) PCL fibers are electrospun onto aluminum foil on a rotating mandrel. *D* = distance between needle tip and mandrel and R = rotational speed of mandrel. The fibers and foil are removed and rotated through the desired angle. (B) The second layer of fibers is then deposited on top the first layer. *θ* = fiber angle. (C) The electrospun scaffold is then placed between two silicone gaskets. The device is then placed onto the base of a non-treated 6-well polystyrene plate. *(D)* Final set up with fibers held in place in the capture device. (E) The collagen hydrogel containing NHDFs is then pipetted into the window which contains the PCL scaffold, creating a hydrogel/electrospun composite scaffold. (F) 60° PCL scaffold combined with a hydrogel. Scale bar = 2 mm.

The 0° bundled fibrous scaffold was achieved by using the following parameters: 0.8 mm ID needle; 21 cm between the needle tip and mandrel; 4 ml h^−1^ flowrate for 3 h; +12 kV; −4 kV; 2000 RPM mandrel speed; and 5 mm s^−1^ needle tip axial velocity. The electrospun fibers were collected onto aluminum foil wrapped around an 8 cm diameter mandrel (figure 1(A)). These parameters meant that the polymer/solvent solution changed from Ohmic flow to convective flow approximately 2 cm before the mandrel. This small distance meant the fibers (each individual fiber diameter = ~3.5 *μ*m) were deposited into bundles as opposed to individual fibers, which structurally differentiates these scaffolds from the majority of fibrous scaffolds found in the literature.

The 30°, 60° and 90° fibrous scaffolds were electrospun using the same parameters as the 0° scaffold, with one exception, after 1.5 h (halfway point), the aluminum foil was lifted and rotated on the mandrel through the desired angle (30°, 60° or 90°) to allow for the second layer of fibers to be deposited along a different axis (figure 1(B)).

The electrospinning set-up was not equipped with temperature and humidity control, which are two environmental factors that are known to affect electrospinning. Therefore, all scaffolds were manufactured within 2 weeks of each other to ensure maximum consistency in the electrospinning process between each group.

### 2.2. Scanning electron microscopy

Acellular PCL scaffolds were visualized using a TM4000 Scanning Electron Microscope (SEM) (Hitachi) with a 15 kV accelerating voltage and a 10 mm working distance. These acellular scaffolds did not require sputter coating prior to visualization.

Engineered tissues allotted for SEM imaging were cultured for 48 h before SEM sample preparation. Tissues were fixed in 4% paraformaldehyde (PF) (MilliporeSigma) for 30 min, replaced with fresh PF every 15 min. After fixation, the tissues were washed three times with phosphate buffer solution (PBS). The tissues were then incubated with increasing concentrations of 200-proof ethanol diluted in Deionised (DI) water. Ethanol concentrations increased in 10% vol increments (10%, 20%… 100%) with each incubation lasting 15 min. The last incubation step of 100% 200-proof ethanol was repeated three times to ensure complete infiltration of the engineered tissues with ethanol. The engineered tissues were kept in 200-proof ethanol at 4 °C for short-term storage.

The engineered tissues underwent critical point drying (Ladd Research Industries, Brown University Leduc Bioimaging Facility) and sputter coating with a deposition current of 20 mA (Emitech K550 Sputter Coater, Brown University Leduc Bioimaging Facility) prior to SEM imaging. The engineered tissues were visualized using a Thermo Apreo VS SEM (Thermo, Brown University Leduc Bioimaging Facility) with a 2 kV accelerating voltage and a 15 mm working distance.

### 2.3. Fiber angle measurements

The angle between the two layers of electrospun fibers on each scaffold was measured using the acellular SEM images to confirm the accuracy of the predicted angles manufactured during the electrospinning process. Briefly, the images were imported into ImageJ, where the angle between fibers on each layer of the scaffold were measured, *N* ⩾ 10. For simplicity, the scaffolds are referred to using their predicted angles and not their actual measured angles.

### 2.4. Fiber diameter, bundle diameter and bundle gap width measurements

**Figure 2.**
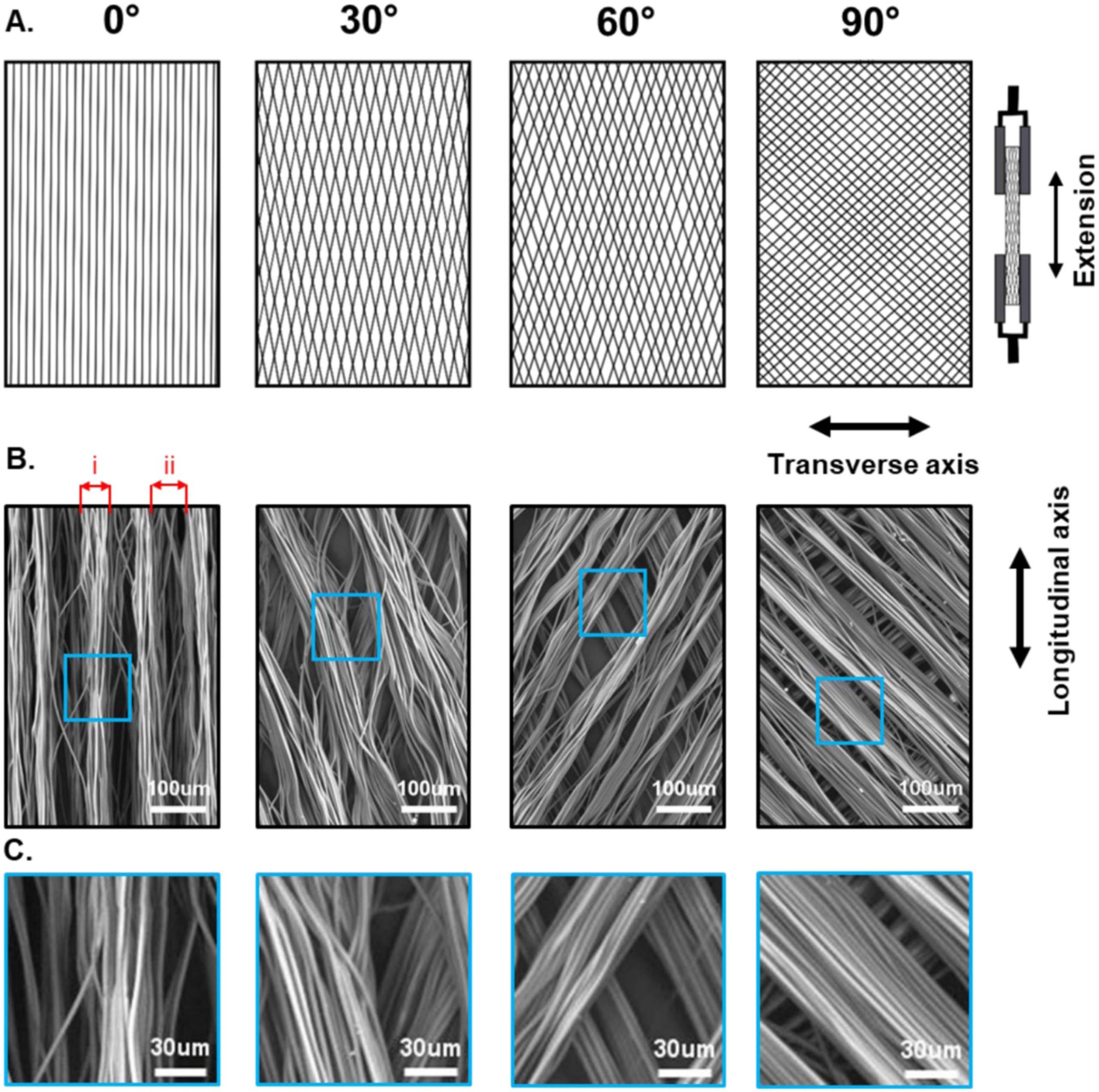
Schematic and SEM imaging showing scaffold architecture and method of tensile assessment. (A) Schematic showing the direction of tension along the longitudinal *(L)* and transverse *(T)* axes. (B) SEM images of each scaffold showing alignment of fiber bundles at differing angles, with (i) showing how bundle diameter and (ii) bundle gap width were measured in table 1. (C) Magnified SEM images showing no welding between layers.

Fiber diameter, bundle diameter and bundle gap width measurements were undertaken using the acellular SEM images imported into ImageJ (figure 2(B)), *N* ⩾ 10.

### 2.5. Tensile testing

Tensile properties of the electrospun acellular scaffolds were measured using two different Instron testing rigs. The first set up was an Instron 3367 loaded with a 50 N load cell. The second set up was an Instron 5940 equipped with a 500 N load cell. Briefly, 30 mm × 5 mm strips of scaffold were cut out and stretched at 10 mm min^−1^ with a starting gauge length of 10 mm. Strips were cut out along the primary and secondary axes of the scaffolds, as shown in figure 2(A). This allowed for the biaxial properties of the scaffolds to be assessed.

The tensile properties of the composite engineered tissues were measured utilizing an Instron 5943 with a 5 N load cell. Conditions for tensile testing of the engineered tissue constructs followed the protocol performed on the acellular constructs.

Young’s modulus was calculated at the steepest point on the stress vs strain curve using the formula:

$$E=\frac{\sigma }{\varepsilon }=\frac{FL_{0}}{A\Delta L}$$

where *E* is the Young’s modulus, *σ* is stress, *ε* is strain, *F* is force, *L*_0_ is the original length, *A* is the cross-sectional area and Δ*L* is the change in length. The elastic limit strain was measured as the point where the linear elastic curve transitioned into plasticity. The elastic limit stress was the stress value at this elastic limit strain. Incremental Young’s moduli were calculated for four strain bands (0%–5%, 5%–10%, 10%–15% and 15%–20%) by measuring the gradient of the stress vs strain curve between the two strain values.

### 2.6. Manufacturing composite scaffolds

#### 2.6.1. Cell culture conditions

NHDFs (a gift from Dr Jeffrey Morgan) were cultured in conditions as previously described [33]. Briefly, NHDFs were cultured on treated tissue culture plates and maintained in Dulbecco’s Modified Eagle’s Medium (DMEM) supplemented with 10% fetal bovine serum (FBS) and 1% penicillin-streptomycin (pen-strep). The NHDFs were expanded and passaged using 0.05% trypsin in versene (0.5 mM Ethylenediaminetetraacetic acid (EDTA) (MilliporeSigma) and 1.1 mM d-glucose (MilliporeSigma) in PBS) and used between passage 9 and 10.

iCell ECs (Fujifilm, Cellular Dynamics) were cultured on gelatin-coated tissue culture plates and maintained in endothelial growth medium 2 (EGM-2; Lonza) and passaged with 0.05% trypsin (Life Technologies) in versene (0.5 mM EDTA (MilliporeSigma) and 1.1 mM d-glucose (MilliporeSigma) in PBS) [34]. The ECs utilized in the experiments were at passage 2.

#### 2.6.2. PCL fiber coating

PCL scaffolds measuring 9 mm × 15 mm were cut from the electrospun PCL sheet and used for cell seeding. Prior to cell seeding, the PCL scaffolds underwent a 25 min incubation in 70% ethanol. Once dry, the scaffolds were washed three times in PBS. The scaffolds were then incubated overnight in either PBS or DMEM media supplemented with Corning^™^ Matrigel^™^ (Fisher, CB-40 230) at a 1× dilution. The scaffolds were kept in a humidity chamber at 4 °C until use. Contact angle measurements (data not shown) were performed to confirm incubation of the PCL scaffolds with DMEM media supplemented with Corning^™^ Matrigel^™^ increased the hydrophilicity of the PCL.

#### 2.6.3. Fabrication of polydimethylsiloxane (PDMS) frames and capture of PCL scaffolds

Custom capture frames were constructed using PDMS. PDMS was prepared according to the instructions of the manufacturer (10:1, base:curing agent). The PDMS solution was casted over a polystyrene surface to achieve a uniform PDMS sheet of approximately 1.4 mm. The PDMS sheet was cured overnight in a 60 °C oven. After curing, 15 mm × 20 mm capture frames were cut out of the PDMS sheet and a 3 mm × 9 mm window was then cut from each 15 mm × 20 mm capture frame. All PDMS frames were autoclaved before use with cells.

Two PDMS capture frames were utilized to secure the PCL scaffold (figures 1(C) and (D)). One frame was positioned at the bottom of a non-treated 6-well polystyrene plate. No adhesive was necessary to secure this frame, likely due to the hydrophobic interaction between the PDMS and polystyrene surface. The PCL scaffold was carefully placed on top of the frame to ensure proper fiber orientation within the window and to avoid folding the PCL scaffold. A small amount of DOWSIL^™^ 732 Multi-Purpose Silicon Sealant (Dow Inc.) sealant mixed with 70% ethanol at a 1:1 volume was applied to the extra PDMS frame space (15 mm × 20 mm as compared to the 9 mm × 15 mm PCL). This created a seal around the edge of the PDMS frames in order to ensure no solution leakage. The second PDMS frame was applied on top the PCL scaffold and first PDMS frame and the sealant was allowed to set.

#### 2.6.4. Preparation of composite engineered tissue

Composite engineered tissues were prepared by combining collagen hydrogel at a concentration of 1.0 mg ml^−1^ with NHDFs and in some cases ECs.

Preparation of the collagen hydrogel has been previously described in detail [35–37]. Briefly, a collagen precursor solution with a concentration of 2.0 mg ml^−1^ collagen was generated utilizing 3.8 mg ml^−1^ rat collagen I commercial stock solution (Advanced Biomatrix, San Diego, CA). The commercial stock solution was diluted with DI water and 10× RPMI 1640, neutralized with 1 M sodium hydroxide in order to achieve a pH of 7.4 and further stabilized with HEPES (4-(2-hydroxyethyl)-1-piperazineethanesulfonic acid) buffer (Sigma, H0887–100ML). All preparation of the collagen hydrogel precursor solution was performed on ice prior to cell retrieval. NHDFs and ECs were obtained through passaging as described previously in section 2.6.1. Cell suspension solutions were diluted in the appropriate media to achieve a concentration of 100 000 cells/ 70 *μ*l.

Immediately before casting, the collagen precursor solution and cell suspension solution were mixed at a 50/50% volume ratio. For each engineered tissue, 70 *μ*l of collagen-cell solution was casted into the PDMS capture frame window and allowed to set for 45 min at 37 °C to ensure proper gelation of the hydrogel (figures 1(E) and (F)). For the constructs composed of both NHDFs and ECs, each cell type was casted in its own collagen hydrogel precursor solution. This way, 35 *μ*l of NHDF cell-collagen solution was pipetted on the top of the PCL scaffold and 35 *μ*l of the EC cell-collagen solution was pipetted on the bottom of the PCL scaffold. The first cell-collagen solution was casted and allowed to gel before the second cell-collagen solution was casted. After gelation, NHDF-only engineered tissues were supplied with DMEM supplemented with 10% fetal FBS and 1% pen-strep while NHDFs and ECs engineered tissues were supplied with 50/50% vol of DMEM supplemented with 10% fetal FBS and 1% pen-strep media and EGM-2 media.

All composite engineered tissues were cultured in an incubator with temperature (37 °C) and carbon dioxide (5%) control for 7 d, at which point subsequent mechanical testing and/or immunofluorescence imaging took place. One exception to this time point is the engineered tissues utilized for SEM imaging, which were collected after 48 h in culture. During culture, the appropriate maintenance media for the engineered tissues was changed every other day and the tissues were monitored daily through brightfield optical microscopy (Olympus SZ40).

### 2.7. Immunofluorescence staining and imaging of the composite engineered tissues

Whole-mount immunohistochemical staining was performed on the composite engineered tissues based on the protocol developed by Kant *et al* [34]. Whole-mount histology was performed in order to maintain the structural integrity of the PCL fibers and cells, thus overcoming the limitations of other techniques, specifically melting of PCL during paraffin embedding due to the low melting temperature of PCL and specimen shearing observed with frozen block cryoembedding and slicing. Immunofluorescence staining targeted Hoescht, vimentin and cluster of differentiation 31 (CD-31).

Composite engineered tissues were fixed in 4% PF (MilliporeSigma) for 10 min then washed and stored in PBS. NHDF-only tissues were blocked with 5% normal goat serum (NGS), 1% Bovine Serum Albumin (MilliporeSigma) and 0.1% Triton-X (MilliporeSigma) in PBS for 2 h. The blocking buffer used for tissues with NHDFs and ECs were devoid of NGS. Following the 2 h incubation in blocking buffer, the engineered tissues were incubated with the primary antibody mouse monoclonal anti-vimentin (V6630–100 UL; MilliporeSigma) at a dilution of 1:200 in fresh blocking buffer (MilliporeSigma). For the tissue constructs with NHDFs and ECs, an additional primary antibody was utilized, rabbit anti-CD31 (ab28364; Abcam) at a dilution of 1:100 in blocking buffer devoid of NGS.

On the second day, all tissues underwent two 2 h PBS washes, 1 h incubation of the appropriate blocking buffer and overnight incubation with secondary antibodies. All tissues were incubated in the secondary antibody goat anti-mouse Alexa Fluor 488 (A-11 001; Invitrogen) at a 1:400 dilution in blocking buffer. For the tissue constructs with NHDFs and ECs, an additional secondary antibody was utilized, goat anti-rabbit Alexa Fluor 594 (A-11 005; Invitrogen) at a 1:400 dilution in blocking buffer devoid of NGS.

On the third and final day, the tissues underwent two PBS washes, treatment with bisbenzimide H-33 342 trihydrochloride (Hoechst; MilliporeSigma) and two 1 h PBS washes. The stained tissues were stored at 4 °C until imaged using an Olympus FV3000 Confocal Microscope (Olympus, Brown University Leduc Bioimaging Facility). All washes and incubation steps were performed at room temperature on an orbital shaker.

### 2.8. NHDFs nuclear alignment analysis

Nuclear alignment of the NHDFs cultured within the composite tissue was determined through the isolation, binarization and watershed segmentation of the Hoechst immunofluorescence channel in MATLAB^®^. Overlapping nuclei, which resulted from out of plane nuclear stains, were removed from analysis as they prevented accurate measurements of nuclear alignment. The major and minor axes of the nuclei were identified and quantified relative to the PCL fiber orientation. Careful measures were taken to maintain directionality of the engineered tissue during the immunofluorescent staining and imaging process. Brightfield images were also obtained on the Olympus FV3000 Confocal Microscope (Olympus, Brown University Leduc Bioimaging Facility) during immunofluorescent imaging to obtain a reference for the PCL fiber angle. Nuclei angle was calculated relative to the PCL fiber. Three or more regions of interest (*N* ⩾ 3) were analyzed for each of the architected engineered tissues to determine nuclear alignment.

### 2.9. Statistical analysis

All statistical analysis was performed in JMP^®^ Pro 15 (SAS Institute Inc, Cary, NC). One-way analysis of variance with Tukey’s post hoc multiple comparison analysis was performed to determine statistical significance. *p* < 0.05 were considered statistically significant. All error bars represent standard deviation.

## 3. Results

### 3.1. Electrospun fiber properties

We began by manufacturing electrospun fibers with four different defined morphologies. Fibers were electrospun in bundles with controlled angles between both layers of the scaffold. Layers were achieved by rotating the bundled electrospun fibers through a set angle and depositing a second layer of aligned fibers on top, as seen in figure 1(A). Briefly, bundled fibrous scaffolds with predicted angles of 0°, 30°, 60° and 90° between the two layers of the scaffold were electrospun (actual angles were measured after electrospinning), with distinct pores between each bundle of fibers, as seen in the SEM images in figure 2(B). The actual angles achieved during electrospinning are shown in table 1. In all cases, the actual angles (0.7°, 35.8°, 56.2° and 88.4°) all ended up being close to the anticipated target angles (0°, 30°, 60° and 90°), with significant differences in the angle noted between all four scaffolds (*p* < 0.001). Scaffold fiber diameters (table 1) were assessed to ensure that no large variances can be found across the different scaffolds. There were no significant differences between the diameters of all four scaffolds (*p* = 0.058–0.995), which ranged from 3.44 *μ*m in the 60° scaffold to 3.87 *μ*m in the 90° scaffold. Furthermore, no obvious welding could be seen between the two layers of the scaffold from SEM images (figure 2(C)).

The average bundle diameters were measured and found to range from 35.8 *μ*m in the 60° scaffold to 62.5 *μ*m in the 90° scaffold, as seen in table 1. The only significant differences noted were between the 60° scaffold and the 0° and 90° scaffolds (*p* = 0.014 and 0.000, respectively). The average gap width between the bundles was controlled by using a 5 mm s^−1^ needle tip axial velocity to ensure that the space was sufficient for adequate infiltration of cardiomyocytes during seeding. For example, cardiomyocytes derived from human induced pluripotent stem cells (hiPSCs) have approximate dimensions of 50 × 25 × 5 *μ*m (cell volume of approximately 5600 *μ*m^3^) [38]. In contrast, mature adult cardiomyocytes have approximate dimensions of 150 × 20 × 15 *μ*m (cell volume of approximately 40 000 *μ*m^3^) [39]. All four scaffolds have gap widths sufficiently large to allow for hiPSC-cardiomyocyte infiltration, as seen in table 1.

### 3.2. Mechanical analysis of electrospun fibrous sheets

Scaffolds were analyzed in tension with a 50 N load cell (Instron 3367) along their longitudinal and transverse axes, as show in figure 2(A). These measurements across the two principal axes were used to assess the anisotropic properties of the scaffolds.

**Figure 3.**
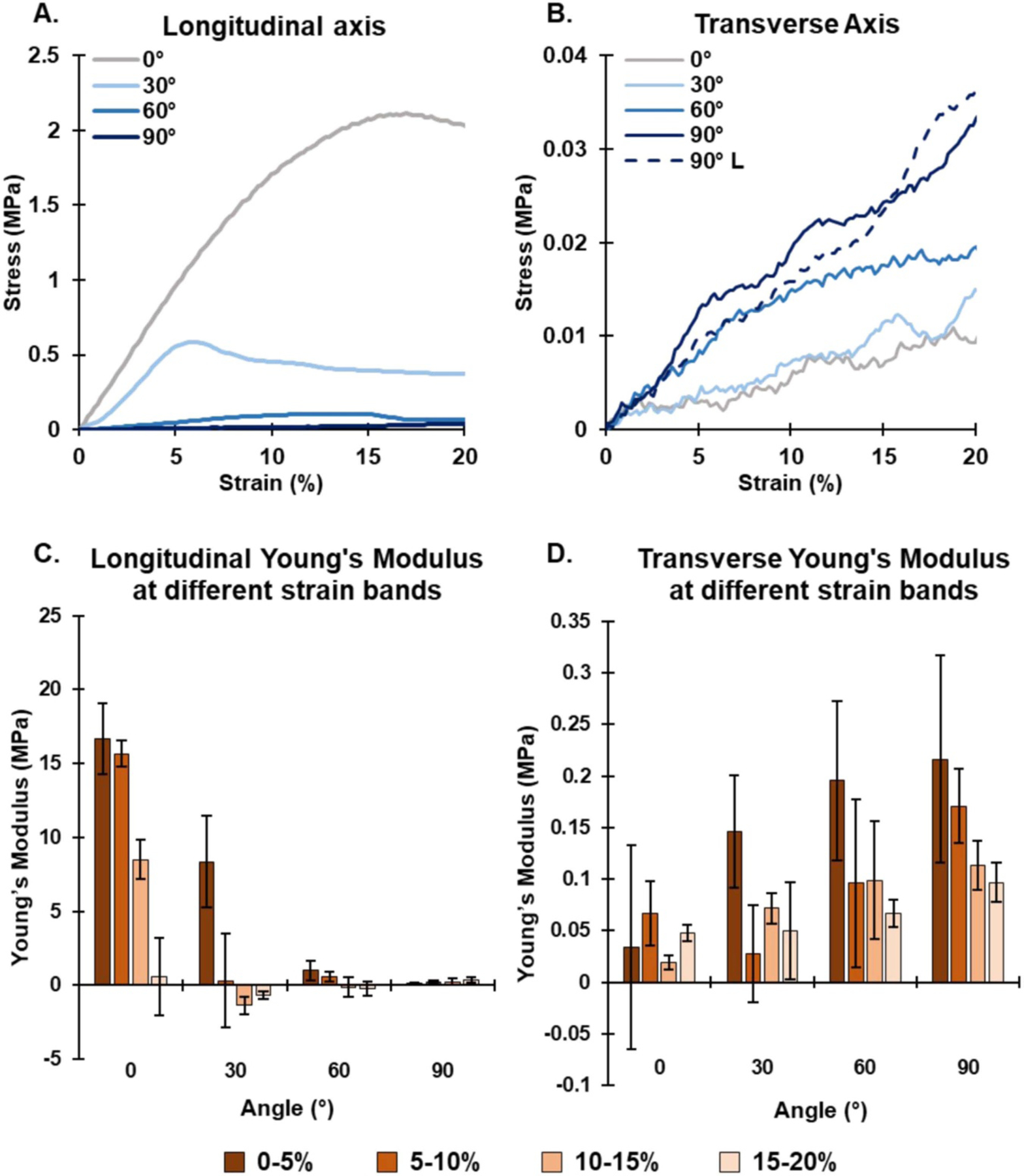
Tensile analysis of architected scaffolds. Representative stress vs strain curves (0%–20% strain region) for all four scaffolds along their (A) longitudinal and (B) transverse axes. The 90° longitudinal curve is also shown on the transverse axis graph (B). Incremental Young’s moduli from 0% to 20% strain for the (C) longitudinal axis and (D) transverse axis, *n* = 3.

Representative stress vs strain curves show the 0%–20% strain region for all four scaffolds along their longitudinal and transverse axes (figures 3(A) and (B)). Incremental Young’s moduli for all four scaffolds along their longitudinal and transverse axes were calculated for 0%–5%, 5%–10%, 10%–15% and 15%–20% strain as shown in figures 3(C) and (D), respectively. The drop in Young’s modulus is steeper in the 0° and 30° scaffolds along their longitudinal axis than the 60° and 90° scaffolds. In contrast, all three scaffolds behave in a similar way along their transverse axis, whereby Young’s modulus declines at a slower rate as strain increases.

**Figure 4.**
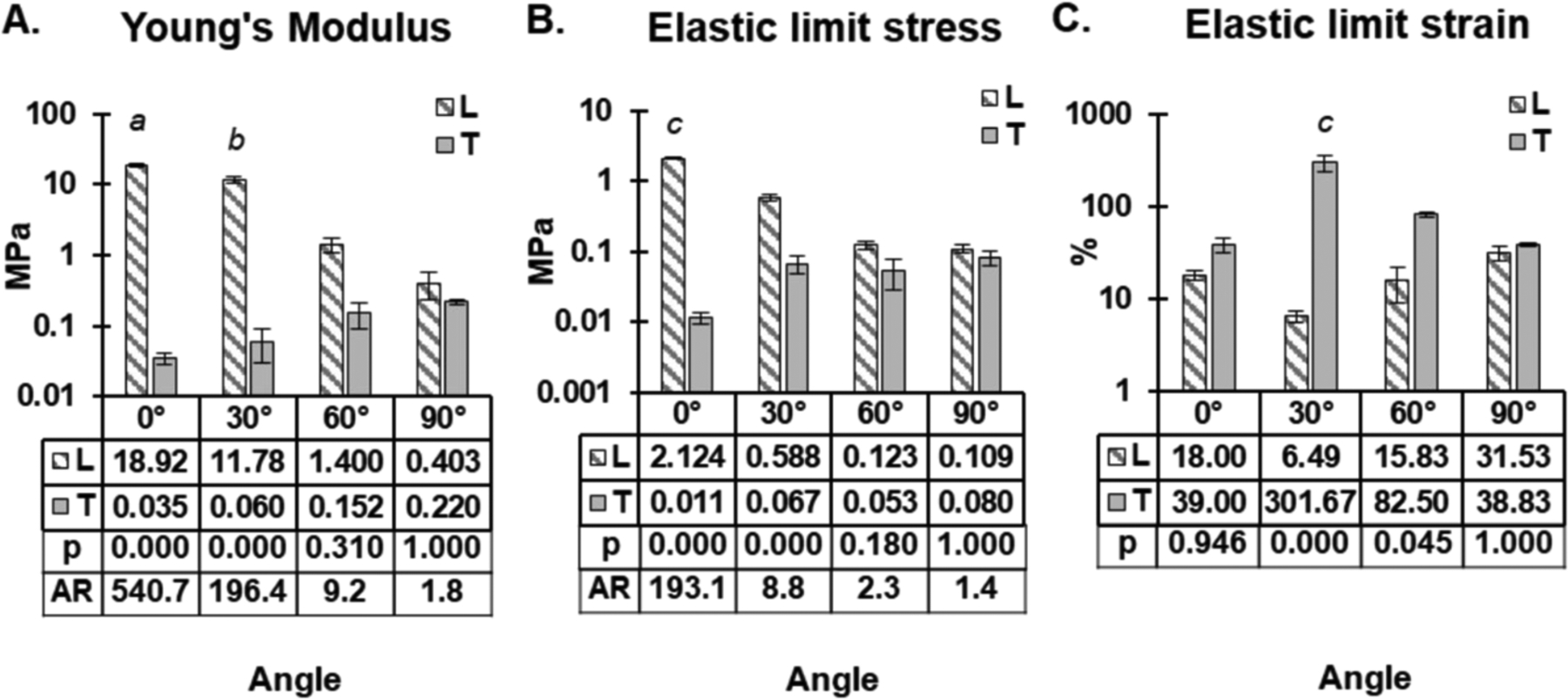
Bulk tensile mechanical properties of architected scaffolds. (A) Young’s modulus of scaffold below 20% strain. (B) Elastic limit stress and (C) the elastic limit strain of the four scaffold along their longitudinal *(L)* and transverse *(T)* axes. Mean values and *p* values can be found in the table below each graph. AR = anisotropic ratio of values between longitudinal axis and transverse axis. Log axes were used to ensure all data could be visualized. *a* and *b* = significantly different to all other values apart from each other. *c* = significantly different to all other values, *n* = 3.

The bulk Young’s modulus of each scaffold was calculated in the 0%–20% strain region using the steepest gradient of the stress vs strain curve. This strain region was chosen as this is the region that is physiologically relevant in cardiac tissue, as seen in figure 4(A). As expected, the Young’s modulus is highest along the longitudinal axis of the 0° scaffold (18.93 ± 0.84 MPa), declining with each scaffold as the angle increased up to 90° (0.40 ± 0.16 MPa), and further dropping back to the 0° scaffold (0.035 ± 0.007 MPa) when the scaffolds were assessed along their transverse axes. This suggests that a smaller angle between the axis of tension and the direction of fiber alignment results in a higher Young’s modulus. Furthermore, these results clearly display the anisotropic properties of the scaffold with regards to Young’s modulus. The anisotropic ratios of longitudinal to transverse Young’s modulus were found to be 540.7, 196.4, 9.2 and 1.8 for the 0°, 30°, 60° and 90° scaffolds, respectively.

Similar results were noted for the stresses at the elastic limit (the point where the scaffold is deemed to have transitioned from elastic deformation to plastic deformation), as seen in figure 4(B). The elastic limit stress was highest along the longitudinal axis of the 0° scaffold (2.12 ± 0.04 MPa), and similar to the Young’s modulus measurements, decreased with each scaffold as the angle increased to 90° (0.11 MPa), and further declined from the 90° scaffold (0.08 ± 0.02 MPa) back to the 0° scaffold (0.011 ± 0.002 MPa) when the scaffolds were assessed along their transverse axes. The anisotropic ratios of longitudinal elastic limit stress to transverse elastic limit stress were found to be 193.1, 8.8, 2.3 and 1.4 for the 0°, 30°, 60° and 90° scaffolds, respectively.

The elastic limit strain results (the strain at which the scaffold was deemed to have transitioned from elastic deformation to plastic deformation) showed a trend of increasing elastic limit strain as the angle between the direction of tension and direction of the fibers was increased, with the 0° scaffold being the exception, as seen in figure 4(C).

### 3.3. Extrapolation of anisotropic scaffold properties to deduce the required scaffold fiber angle

**Figure 5.**
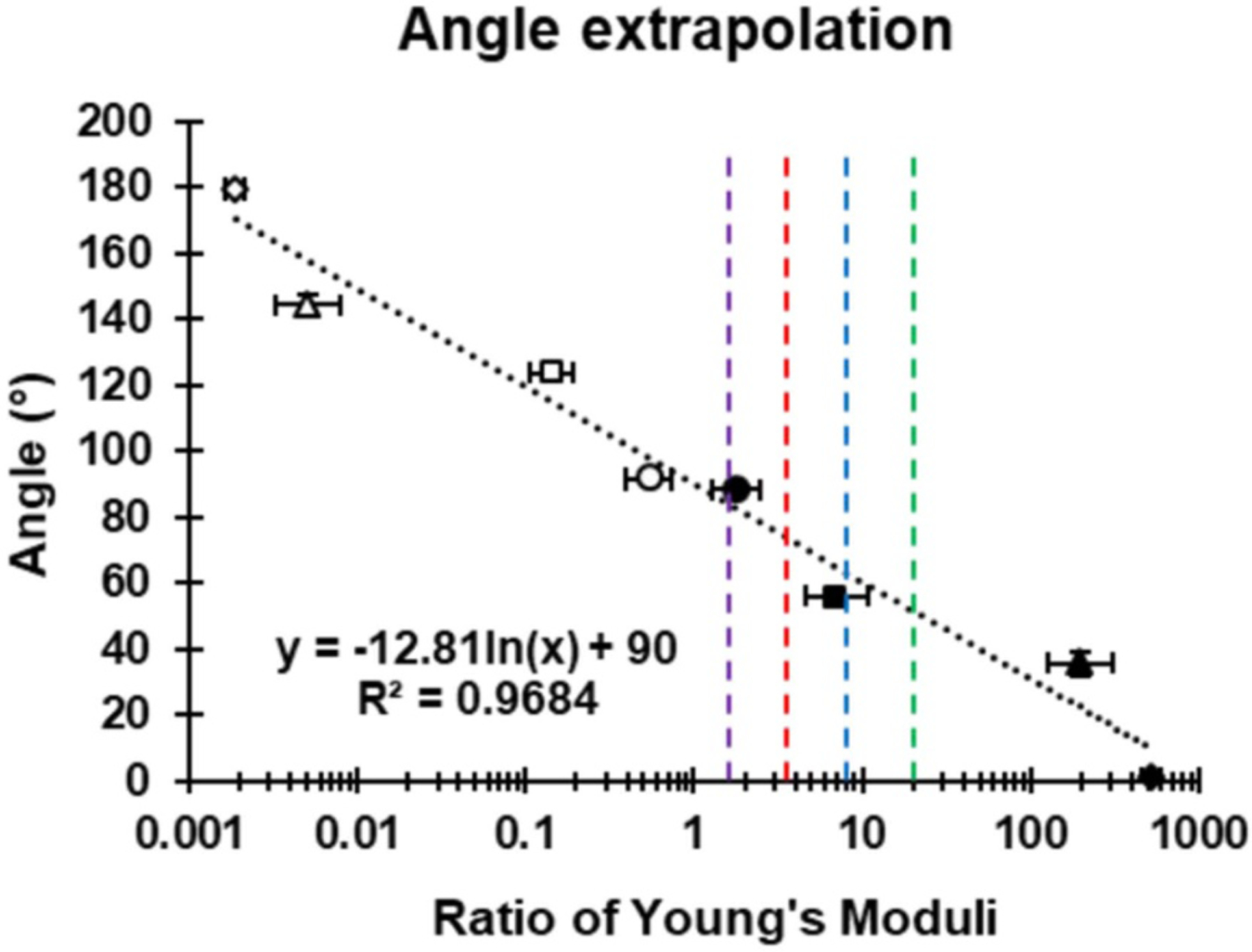
Architected angle extrapolation for the design of scaffolds with desired mechanical properties. Correlation between the anisotropic ratio of Young’s moduli (longitudinal axis and transverse axis) and fiber angle. The equation of the line of best fit can be used to extrapolate the angle required to achieve a desired anisotropic ratio of Young’s moduli. The open symbols were calculated from the same scaffold as their equivalent filled symbols. *Y*-axis error bars are small and in some cases do not extend beyond the symbols. Purple dashed line = human articular cartilage (ratio = 1.47) [40]. Red dashed line = human myocardium (ratio = 3.25) [3]. Blue dashed line = human aortic heart valve (ratio = 7.80) [41]. Green dashed line = human tendon (ratio = 19.41) [3].

The anisotropic ratio of Young’s moduli along each scaffold’s longitudinal and transverse axes were plotted against the fiber angle as a means of extrapolating the required angle of fiber to achieve a desired anisotropic ratio of Young’s moduli, shown in figure 5. A logarithmic line of best fit was plotted with an *R*^2^ value of 0.9684 suggesting a strong correlation between fiber angle and the log anisotropic ratio of Young’s moduli. The equation of the line is:

Angle(°)=−12.81×ln(desiredratioofYoung’sModuli)+90.

This equation can then be used to back calculate the required fiber angle to achieve the desired anisotropic ratio of longitudinal vs transverse Young’s moduli. For example, Kaiser *et al* found that the anisotropic ratio of Young’s moduli in fresh healthy rat myocardium was approximately 1.86 [36]. Therefore, the above equation suggests that a scaffold with fiber angles of 82.05° would most accurately mimic the anisotropic properties of the native rat myocardium. This method of extrapolation can also be used with other anisotropic tissues, as shown in table 2, in order to create custom anisotropic scaffolds for many different applications in healthcare and medicine. Therefore, these novel architected scaffolds can be used in all facets of tissue engineering where anisotropy is present.

### 3.4. Attachment and infiltration of NHDFs within the composite engineered tissue

In order to validate the ability of the proposed scaffold to be used in a variety of tissue engineering applications, NHDFs were cultured on PCL scaffolds. The composite engineered tissues composed of NHDFs were analyzed to determine the ability of the architectured PCL scaffolds to support cell attachment and infiltration, two features in which other synthetic scaffolds often fail [48].

**Figure 6.**
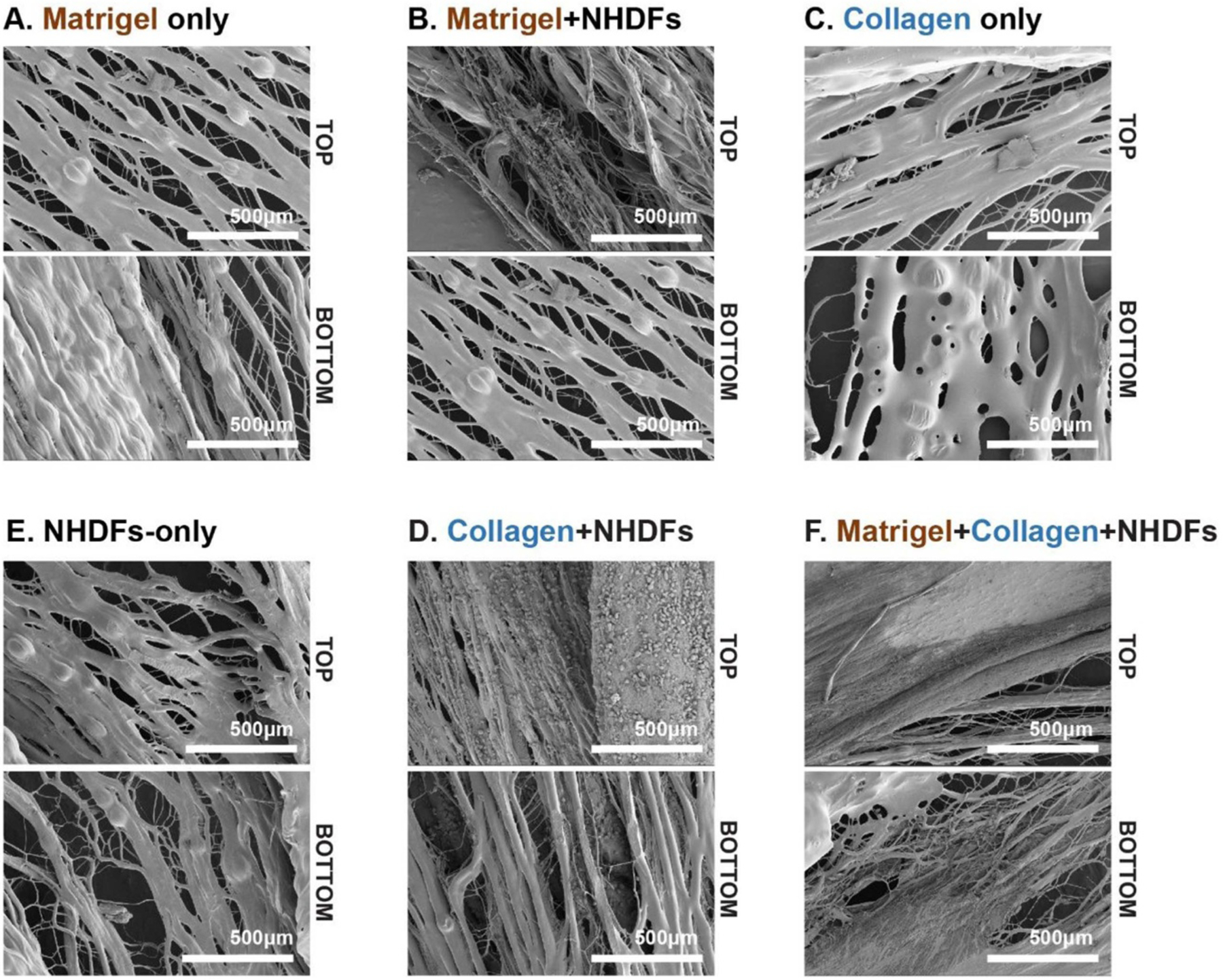
Evaluation of PCL scaffolds treatments with SEM evaluation in order to determine the impact of treatment on NHDFs attachment and infiltration. The treatments of the 0° architectured PCL scaffolds included: (A) 1X Matrigel^™^ in DMEM overnight incubation; (B) 1X Matrigel^™^ in DMEM overnight incubation with NHDFs seeding (100 000 cells/construct); (C) collagen hydrogel (1 mg ml^−1^) seeding; (D) collagen hydrogel (1 mg ml^−1^) with NHDFs seeding (100 000 cells/construct); (E) bare PCL scaffold with NHDF seeding (100 000 cells/construct); and (F) 1X Matrigel^™^ in DMEM overnight incubation and collagen hydrogel (1 mg ml^−1^) casting with NHDFs seeding (100 000 cells/construct). All tissues were evaluated after 48 h in culture. Scale bars = 500 *μ*m.

For these experiments, only 0° architectured PCL scaffolds were evaluated. In addition, infiltration of the cells within the PCL scaffold was determined by maintaining orientation of the top (defined as the face of the scaffold on which cells were pipetted directly on) and bottom (defined as the face the scaffolds which is closer to the tissue culture plate, as described in section 2.6.3 and figure 1) of the PCL scaffold. As illustrated in figure 6, the PCL scaffold underwent different treatments prior to NHDF-seeding. When NHDFs were seeded directly onto the PCL scaffold (figure 6(E)), little cell attachment was observed. This is likely due to the hydrophobicity and lack of cell adhesive sites on PCL [48]. In order to increase the hydrophilicity of the PCL as well as provide a cell adhesive coating, two treatment methods of the PCL scaffold were considered: overnight incubation of the PCL scaffold with 1X Matrigel^™^ in DMEM (figures 6(A) and (B)) and the casting of a 1 mg ml^−1^ collagen hydrogel on the PCL scaffold (figures 6(C) and (D)).

As illustrated in figures 6(A) and (C), treatment of the PCL scaffold with either the Matrigel^™^ solution or collagen hydrogel resulted in a thin coating of the PCL fibers. The PCL scaffolds incubated in the Matrigel^™^ solution had a slightly rougher surface as compared to the scaffold seeded with the collagen hydrogel, likely due to the variety of proteins within the Matrigel^™^. When NHDFs were seeded on these two treated scaffolds, both were able to promote cell attachment, as illustrated by the presence of NHDFs on both the top and bottom of the PCL scaffold (figures 6(B) and (D)). When considering cell infiltration, the PCL scaffolds incubated with only the Matrigel^™^ solution had little cell infiltration through the scaffold, as determined by the lack of NHDFs on the back of the scaffold. PCL scaffolds with the collagen hydrogel, on the other hand, appeared to better promote cell infiltration. However, minimal spreading of the NHDFs were observed on these scaffolds as the NHDFs appear globular and unconnected (figure 6(D)).

PCL scaffolds which underwent both a Matrigel^™^ solution overnight incubation and casting of collagen hydrogel had high levels of NHDFs attachment and infiltration, as illustrated in figure 6(F). Further, the NHDFs formed interconnected cellular sheets which spanned the top and bottom of the PCL scaffold. Therefore, for subsequent studies the PCL scaffolds were treated with both a Matrigel^™^ solution overnight incubation and collagen hydrogel.

**Figure 7.**
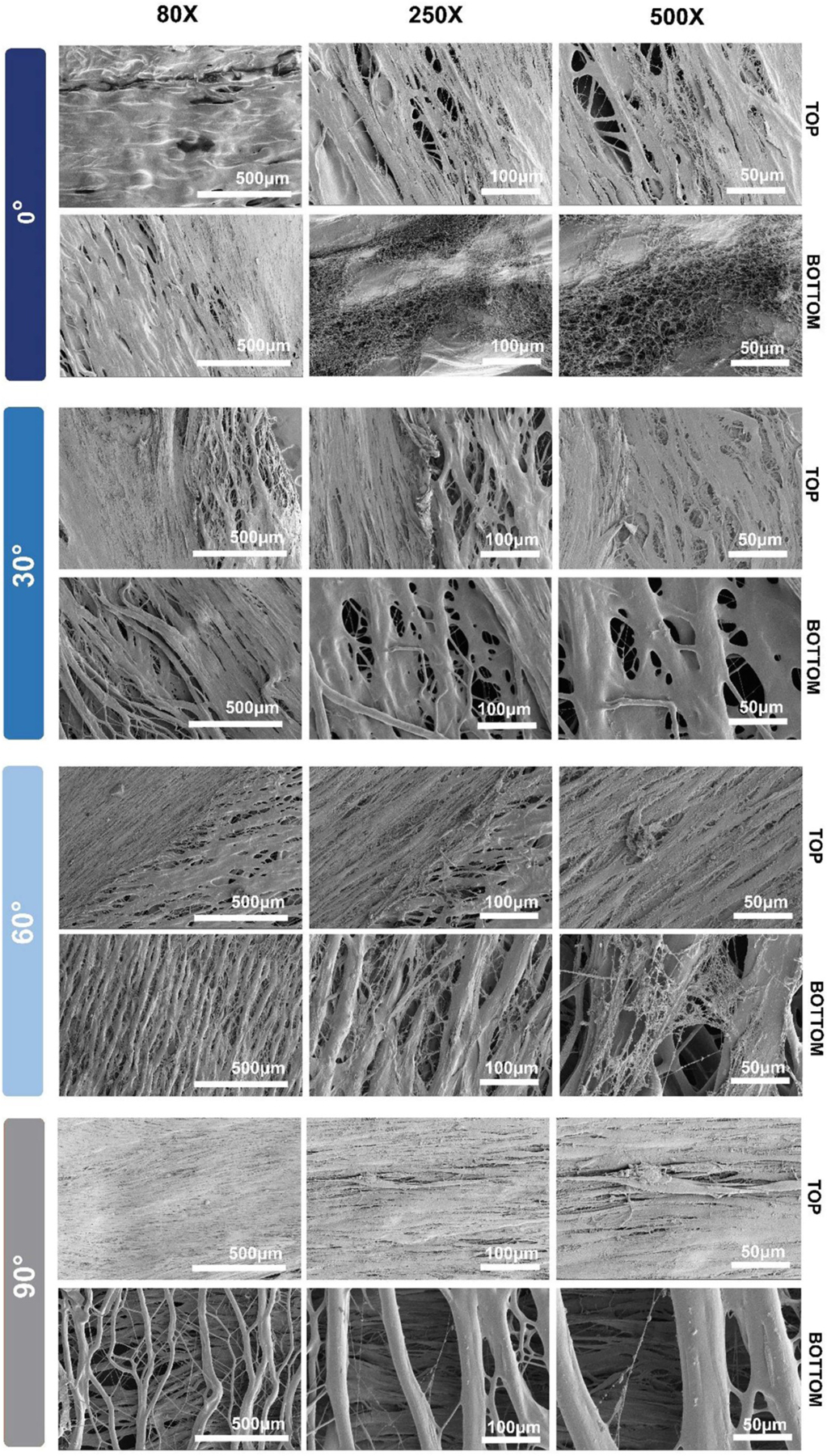
SEM of architectured PCL scaffolds seeded with NHDFs after 48 h. (A) 0° architectured PCL scaffold top and bottom; (B) 30° architectured PCL scaffold top and bottom; (C) 60° architectured PCL scaffold top and bottom; and (D) 90° architectured PCL scaffold top and bottom. Top is defined as the face of the scaffold on which the NHDFs were pipetted directly on and bottom is defined as the face the scaffolds which is closer to the tissue culture plate, on which the NHDFs were not directly seeded on.

NHDFs were then cultured on the different architectured PCL scaffolds for 48 h and evaluated using SEM in order to determine the influence of fiber directionality on initial cell attachment and growth. SEM images of both the top and bottom of the scaffold for every fiber architecture is summarized in figure 7. As expected, the NHDFs were able to attach to the top of the PCL scaffolds for all architectures. In many cases such as 0° top and bottom, 30° top and bottom, 60° top and 90° top, a dense monolayer of NHDFs formed which featured a high density of interconnected, elongated NHDFs. Interestingly, the 90° architectured scaffold featured a dense monolayer on the top face of the scaffold, but had little cell attachment on the bottom face of the scaffold. One explanation for this decreased cell infiltration could be the small bundle gap of the PCL fibers in the 90° architecture (36.6 ± 8.6 *μ*m), which may inhibit complete NHDF infiltration into the scaffold. However, the 60° architectured scaffold did feature NHDFs on both the top and bottom of the scaffold, despite having a similar bundle gap width as compared to the 90° architectured scaffold (35.6 ± 12.0 *μ*m). Even though the 60° architectured scaffold did feature moderate cell attachment, neither the 60° or 90° architectures feature the dense monolayers observed on the back of the 0° and 30° architectures. Therefore, decreased bundle gap width and increased angle of the PCL fibers may be important features that impact the ability and/or speed by which cells can infiltrate the scaffold and could be utilized as tunable parameters based on the requirements for a specific scaffolding application.

### 3.5. Mechanical testing on engineered NDHF tissues

**Figure 8.**
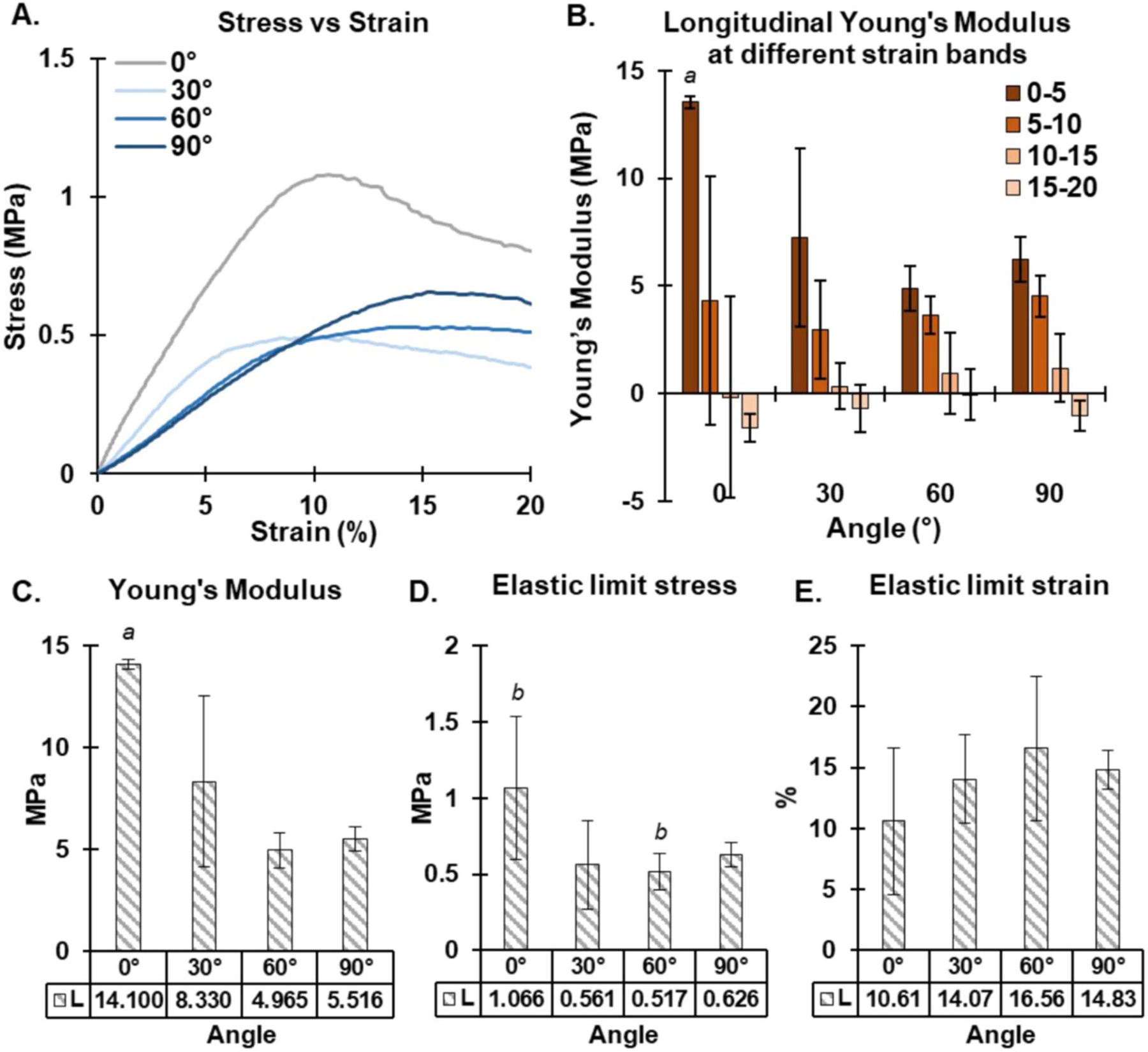
Tensile analysis of architected composite scaffolds. (A) Representative stress vs strain curves (0%–20% strain region) for all four scaffolds. (B) Incremental Young’s moduli for 0%–20% strain along the longitudinal axis. (C) Bulk tensile Young’s modulus of the composite scaffolds below 20% strain. (D) Elastic limit stress and (E) the elastic limit strain of the four scaffolds along their longitudinal axis. *a* = significantly different to all other values, *b* = significantly different to each other, *n* = 4.

Representative stress vs strain curves show the 0%–20% strain region for all four composite scaffolds along their longitudinal axis (figure 8(A)). Incremental Young’s moduli for all four scaffolds along their longitudinal axis were calculated for 0%–5%, 5%–10%, 10%–15% and 15%–20% strain as shown in figure 8(B). The Young’s modulus for the 0° scaffold in the 0%–5% strain region was significantly higher than the Young’s modulus for all the other scaffolds across that same strain range. All four composite scaffolds behaved in a similar way to each other in the 5%–10%, 10%–15% and 15%–20% strain regions.

The bulk Young’s modulus of each scaffold was calculated in the 0%–20% strain region using the steepest gradient of the stress vs strain curve (figure 8(C)). As expected, the bulk Young’s modulus is highest in the 0° composite scaffold (14.10 ± 0.24 MPa) compared to the three other composite scaffolds which were all significantly lower (ranging from 4.97 ± 0.85 MPa to 8.33 ± 4.20 MPa).

Similar results were noted for the stresses at the elastic limit, as seen in figure 8(D). The elastic limit stress was highest in the 0° composite scaffold (1.07 ± 0.47 MPa), and similar to the Young’s modulus measurements, decreased in three other scaffolds (ranging from 0.52 ± 0.12 MPa to 0.63 ± 0.08 MPa), with significance noted between the 0° composite scaffold and the 60° composite scaffold. The elastic limit strain results showed no real difference between the four composite scaffolds with the strain values ranging from 10.61 ± 6.00% to 16.56 ± 5.89%.

### 3.6. Immunofluorescence and nuclear alignment of composite engineered NHDFs tissues

**Figure 9.**
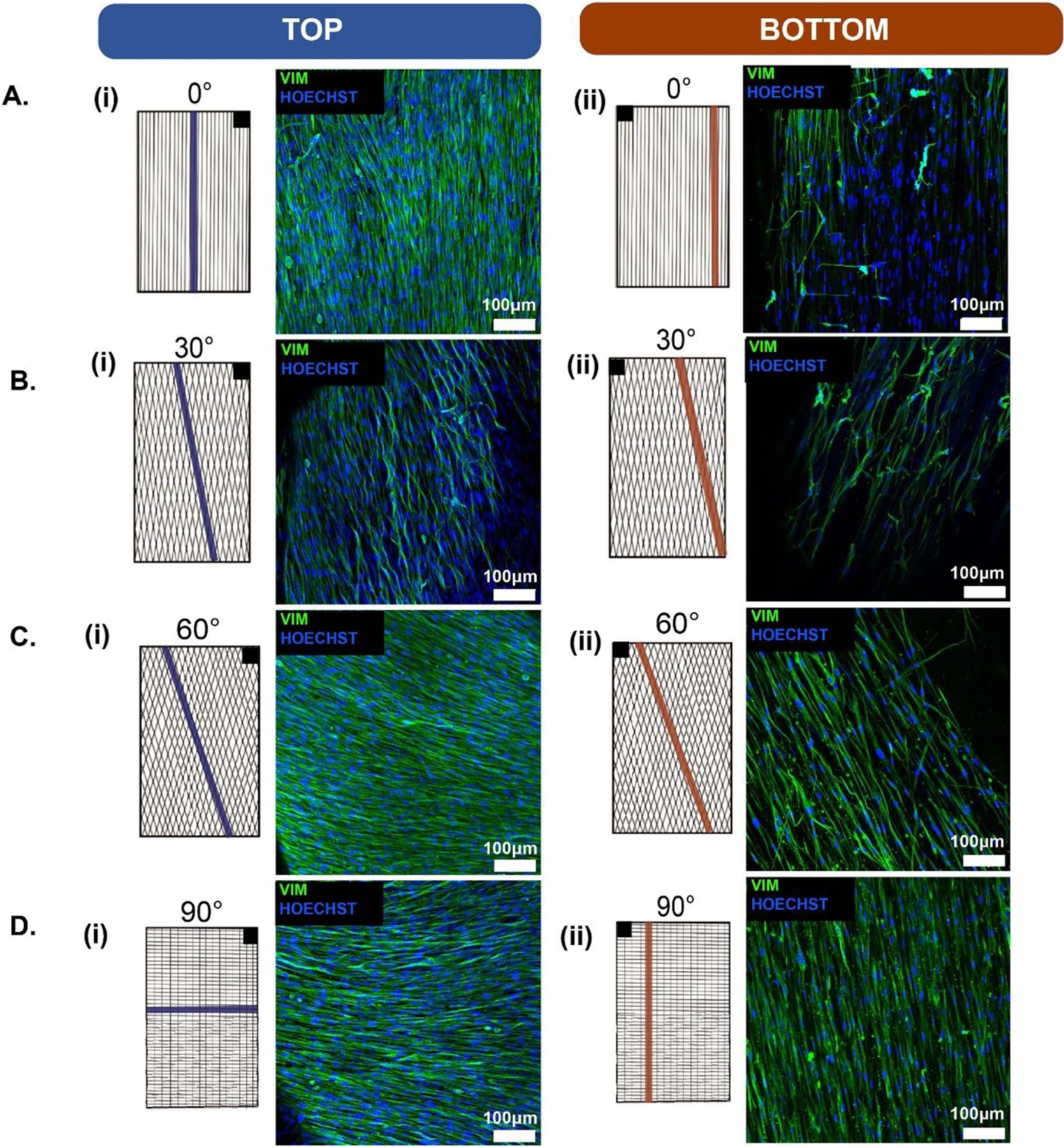
Immunofluorescence of the composite engineered NHDFs tissues for different PCL fiber architectures. (A) 0° architectured PCL scaffold top and bottom; (B) 30° architectured PCL scaffold top and bottom; (C) 60° architectured PCL scaffold top and bottom; and (D) 90° architectured PCL scaffold top and bottom. In order to image both the front and back, the engineered tissue was flipped along its longitudinal axis. The black box in the upper right (i) or upper left (ii) corner of the schematics are intended to illustrate the direction the engineered tissue was flipped for top and bottom face imaging. Scale bars = 100 *μ*m.

In order to determine the impact of PCL fiber architecture on NHDF morphology and elongation, immunofluorescent staining of the engineered tissues composed of NHDFs was performed to visualize vimentin, a type III intermediate filament protein, and Hoechst, a nuclear marker. Representative fluorescent images are illustrated in figures 9(A)–(D). It is clear from these images that the NHDFs encapsulated within the composite engineered tissue align their major axes with the directionality of the PCL fiber. This elongation of the NHDFs along the PCL fiber was not only present on the top side of the scaffold, but was also present on the bottom side of the scaffold for most architecture conditions. This is most notably illustrated in the 90° composite scaffold (figure 9(D)), which features NHDFs following a lateral orientation on the top face of the scaffold (figure 9(D(i))) and NHDFs on the bottom face following a longitudinal orientation (figure 9(D(ii))). However, not all architectured scaffolds could induce such cell alignment on both the top and bottom face. In the 60° composite scaffold, for example, the NHDFs predominantly followed the orientation of the PCL fibers on the top face of the scaffold. Future work is necessary in order to better determine and understand the parameters which impact the ability of the NHDFs to sense the polymer fibers and align themselves accordingly.

**Figure 10.**
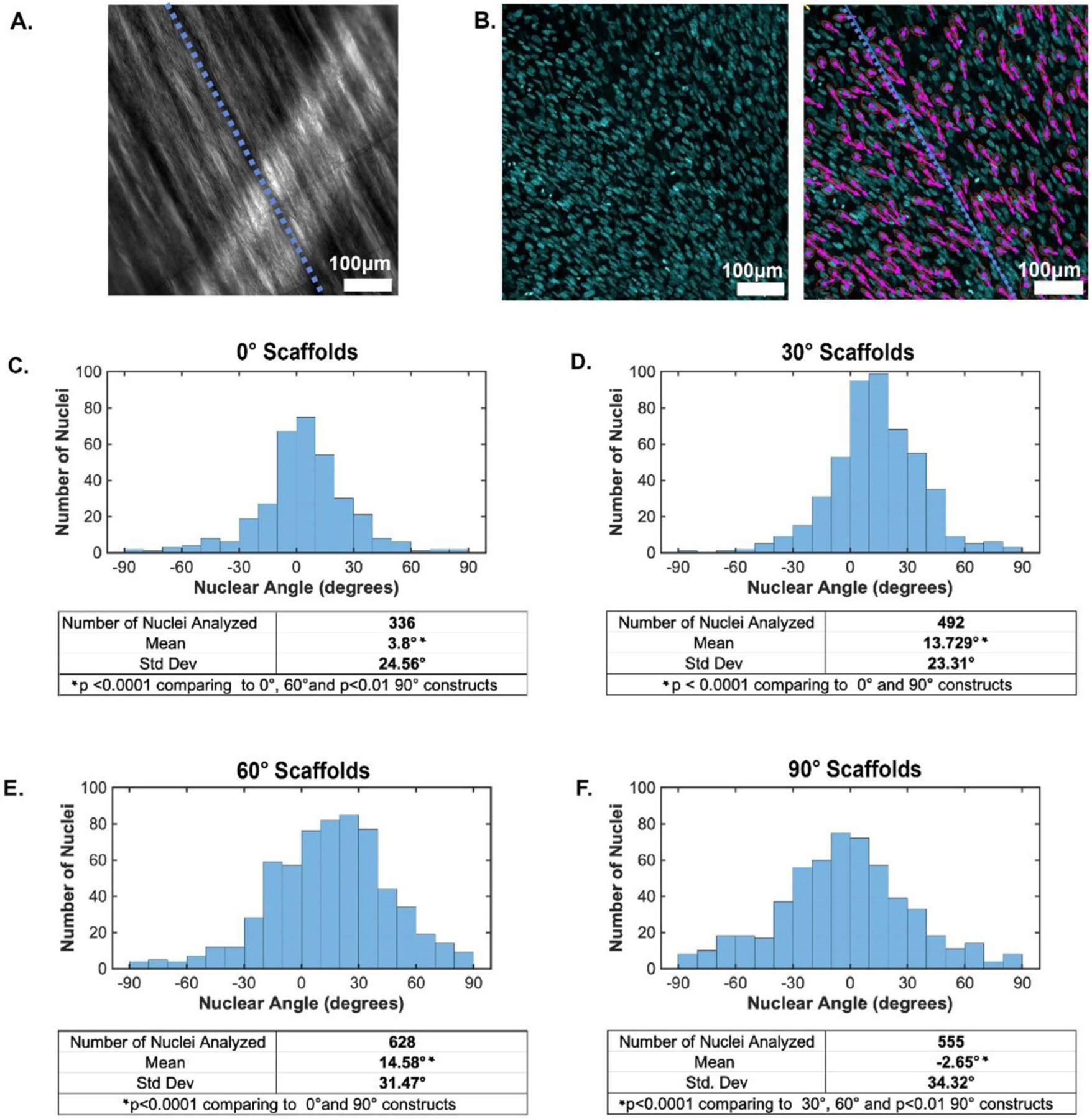
Nuclear alignment analysis of the NHDFs seeded on the architectured PCL scaffolds. (A) and (B) To determine nuclear angle, a reference line along the PCL fiber was determined (blue dotted, (A)) utilizing brightfield imaging of the scaffolds. The Hoechst immunofluorescence channel was isolated ((B), left), binarized and segmented to determine the major and minor axes of the Hoechst-stained nuclei (purple arrows in (B), right) relative to the reference line (blue dotted, (A) and (B), right). (C)–(F) Nuclei angle was plotted as a histogram against the nuclei number for each architected PCL scaffold (C)–(F).

Nuclear alignment analysis was performed to quantify NHDF alignment along the architectured PCL scaffolds. Brightfield images of the PCL fibers were used to generate a reference line which followed the directionality of the PCL fibers for each region of interest within the engineered tissues (figure 10(A), blue line). By isolating the Hoechst fluorescence channel, the major and minor axes of NHDF nuclei were identified (figure 10(B)) and the angle between the PCL fiber and major axis of each nuclei was quantified (figures 10(C)–(F)). In this way, complete alignment of the NHDFs along the PCL fibers would result in the angle between the major axis of the nuclei and PCL fibers to be 0. Positive angle values indicate the directionality of the nuclei is oriented to the right of the PCL fiber while negative angles indicate the directionality is to the left.

Unsurprisingly, the 0° architectured PCL scaffold had a low mean nuclear angle, indicating the NHDFs cultured within this scaffold predominantly followed the orientation of the PCL fibers. Interestingly, however, it was the 90° architectured PCL scaffold which had the lowest nuclear angle mean. Yet, this architecture also had the largest standard deviation, most likely explained by the steep angle gradient between the top and bottom face of the PCL scaffold. Both the 30° and 60° composite engineered tissues had relatively low standard deviations (23.31° and 31.47°, respectively) illustrating the ability of the tested range of architected PCL angles to induce NHDFs nuclear elongation and organization along the specified fiber directionality.

### 3.7. Endothelialization of composite engineered NHDFs tissue

Given the unique composite nature of the engineered system, the viability of two different cell types, specifically NHDFs and ECs, to be cultured on different faces of the PCL scaffold was explored. With endothelization being a vital aspect of injury recovery as well as in vascularization of engineered tissues to overcome the ~150 *μ*m nutrient diffusion limit [49], the ability to culture ECs with NHDFs would greatly enhance the applicability, tunability and specificity of this system.

**Figure 11.**
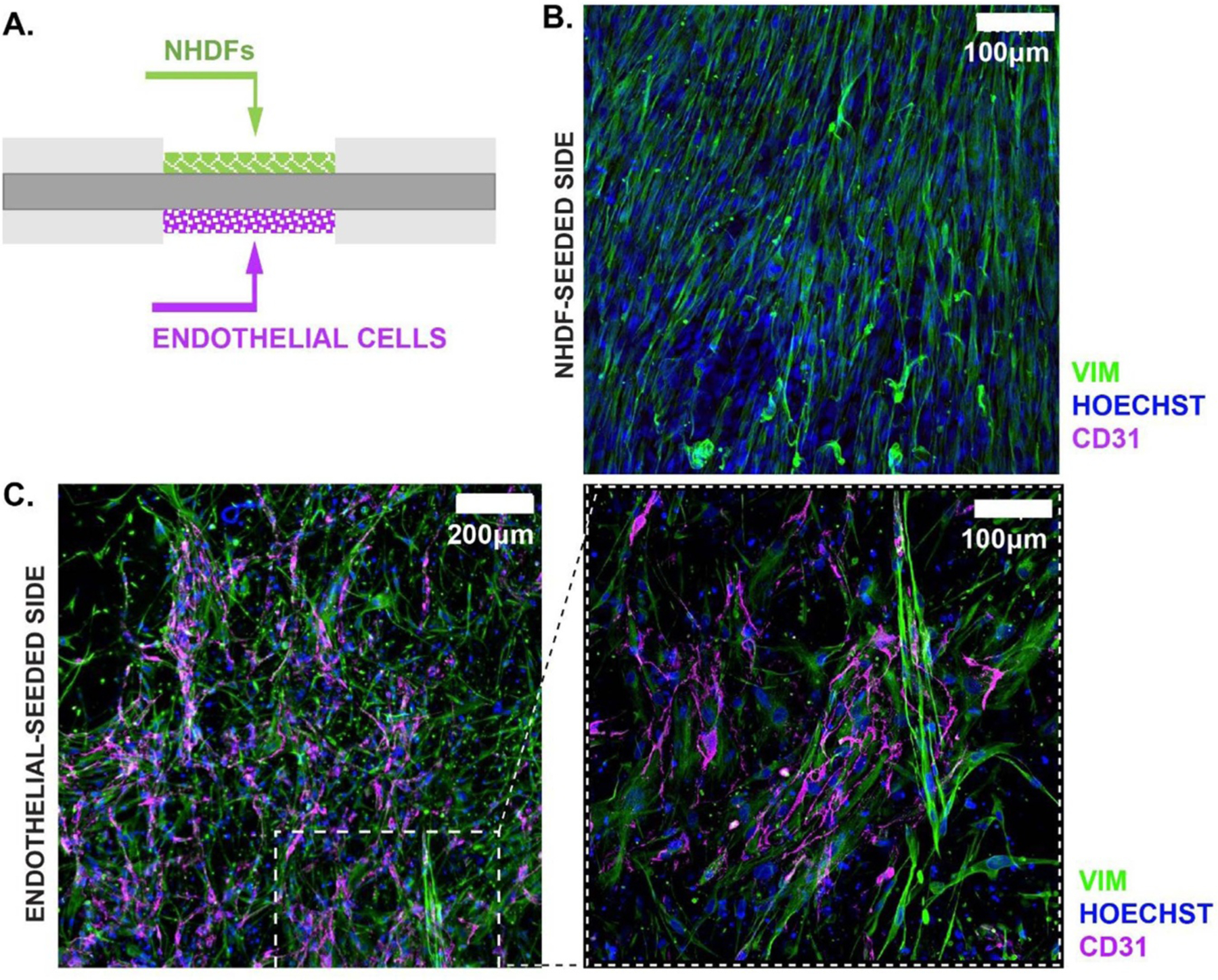
Endothelialization of the NHDFs tissue construct. (A) Schematic for the casting of NHDFs and ECs on the top and bottom of the PCL scaffold. (B) and (C) Immunofluorescent images of the fibroblast-seeded and endothelial seeded side, illustrating localization of the ECs to the bottom of the PCL scaffold.

NHDFs and ECs were cast on different faces of an unaligned PCL scaffold (figure 11(A)). The ECs were seeded first and allowed to properly gel before the seeding of the NHDFs on the other face. Not only did both NHDFs and ECs attach and form a viable engineered tissue, localization of the two different cell types occurred. As illustrated in figures 11(B) and (C) and in the previous experiments, the NHDFs were able to infiltrate through the scaffold as evidenced by their immunofluorescence presence on both the NHDF-seeded and EC-seeded face of the scaffold. However, the ECs were unable to infiltrate the scaffold causing localization of the ECs to only one face (figure 11(C)). One potential explanation is the lower volume used to cast the cells on each face (50 000 NHDFs/35 *μ*l and 50 000 ECs to achieve a total of 100 000 cells/70 *μ*l). Future work modulating cell density will provide further insight into the interaction and localization of multiple cell types within the PCL composite system.

## 4. Discussion

The majority of tissues have a degree of anisotropy in both their physiological and mechanical properties [3, 4]. Mimicking these anisotropic properties is of crucial importance in the field of tissue engineering as these properties drive cell adhesion, differentiation and proliferation [5]. This study developed novel architected scaffolds with anisotropic mechanical properties in an attempt to mimic this inherent anisotropy found in most tissues. Through data extrapolation of mechanical results, any tissue’s anisotropic mechanical properties can be mimicked by electrospinning a bundled aligned fibrous scaffold with layers at different angles to each other. Furthermore, this establishes the basis to develop highly tailored scaffolds and to predict and optimize the mechanical performance of these engineered tissues.

By altering the angle of incidence between each layer of the scaffold, a range of different anisotropic mechanical properties were achieved. Four unique architectures were electrospun with increasing angles between each layer of parallel fibers (0°, 30°, 60° and 90°). The discrepancies between the anticipated target angle and the actual measured angle (table 1) can be attributed to human error when manually rotating the electrospun fibers between the two layers of electrospinning. These four novel scaffolds were manufactured with varying fiber angles and large pores between each bundle of fibers to allow for cellular infiltration. As expected, increasing fiber alignment (decreasing angle) increased the scaffold’s Young’s modulus and its elastic limit stress (figure 4). This phenomena has been seen in many studies looking at the effects of fiber alignment [8, 23, 50–52]. Markatos *et al* also found that increasing fiber alignment (decreasing angle) increased the Young’s modulus in the direction parallel to mandrel rotation [52]. However, unlike the present study, they were unable to maintain similar fiber morphology between each scaffold.

Past studies looking at the role of electrospun fiber alignment in tissue regeneration have focused on extremes of either end—randomly orientated fibers (isotropy) or aligned fibers along one plane (high degree of anisotropy. Our study is the first to mimic the varying degrees of anisotropy found in most tissues, as seen in table 2. We found that the 0° highly aligned fibers had an anisotropic ratio of 540 (Young’s modulus along primary axis is 540 times larger than along the secondary axis.), with their longitudinal Young’s moduli being similar to other electrospun aligned PCL scaffolds [53]. Therefore, there is an argument that an aligned (0°) fiber with highly anisotropic properties is inappropriate for use with any tissue type if mimicking native mechanical properties is deemed necessary. For example, this study found that an angle of 52° would generate a scaffold with the same anisotropy as tendon tissue, which has always been considered a highly anisotropic tissue (table 2), with many studies using highly aligned (0°) fibers [13, 54, 55]. The same idea applies to the use of randomly orientated fibers when dealing with tissues that are often considered isotropic, such as the kidney and skin. A completely random scaffold would have a anisotropic ratio of 1, whereas these tissues have ratios of 1.89 and 1.96, respectively [43, 44]. Therefore, due to the inherent anisotropy found in most tissues, more emphasis should be made on scaffold anisotropy to accurately mimic native tissue properties, such as the novel electrospinning method presented in this study. The electrospinning method used to design these novel scaffolds allows for a range of different angles to be architected throughout the thickness of the scaffold. Many tissues *in vivo* have varying physical structures, with morphological changes found across the tissue to handle variable forces [4, 56]. For example, several groups have studied how myocardial fibers are arranged through the thickness of the heart’s wall (endocardium, myocardium and epicardium). They found that fiber orientation varied gradually through the depth of the ventricular wall, with an angular orientation starting at around—60°/70° at the epicardium rotating progressively to 70°/80° at the endocardium [4, 57–59]. This represents a total angular rotation of approximately 150° across the entire wall’s thickness, which can be architected into the scaffold.

We focused on the 0%–20% strain region for the Young’s modulus as these are typical working strains seen by most tissues [60, 61]. The maximum strains typically found in the myocardium range from 11% to 19.3% depending on the layer being analyzed; with increasing strain as the layer moves towards the outer wall [60, 61]. The testing regimen used in this study was focused around cardiac tissue. Since different tissues undergo a range of different strains and strain rates, the testing regimen would need to be tailored for each tissue type to more accurately mimic their mechanical properties [62]. For example, the anterior cruciate ligaments has been shown to undergo approximately 4%–6% strain during normal function at much higher strain rates than those seen in cardiac tissue [63].

Many studies have shown how different cells are capable of sensing a scaffold’s physical properties such as fiber alignment. It has been shown that cardiomyocytes encapsulated in a hydrogel were capable of sensing the stiffness and direction of the fibrin thread at a distance of up to 100 *μ*m [64]. The fibrous scaffolds presented in this study have average bundle gap widths ranging from 35.6 ± 12.0 *μ*m to 79.6 ± 20.4 *μ*m, meaning that all cells should be close enough to a PCL bundle, ensuring that the local fiber stiffness is transmitted to the seeded cardiomyocytes. Furthermore, cellular phenotypic and genotypic changes have also been noted due to alterations in fiber alignments. Cardiomyocytes grown on aligned electrospun fibers showed increased sarcomere length and increased expression of two genes associated with cardiomyocyte maturation: myosin heavy chain adult isoform (MYH7) and calsequestrin (CASQ2) [12]. Similarly, it has been shown that increasing the alignment of cultured cardiomyocytes increases their expression of myosin heavy chain *α* isoform (MYH6); a key marker for functional contractile cardiomyocytes [24]. Beyond the realm of cardiac tissue engineering, fiber alignment has also been shown to affect several other tissue types on a cellular phenotypic and genotypic level [10, 21, 53, 54]. For example, in skeletal tissue engineering, increasing scaffold fiber alignment led to increased cell lengthening (phenotypic characteristic of healthy skeletal muscle cells) and found significantly increased differentiation of myoblasts into myofibers (required for efficient contraction) [21]. A scaffold’s ability to interact with different cell populations is of critical importance to its long term efficacy as an implant. Seeded cells *in vitro*; and both repopulating cells and immune cells *in vivo* all need to react favorably to the scaffold [65, 66]. In particular, host immune response and repopulating cell response are important considerations when dealing with biological implants [65]. It has been widely reported that implantation of foreign material, including scaffold materials and/or cells, can lead to implant rejection, which would be considered a catastrophic failure [67, 68]. Therefore, the scaffolds presented in this study were designed so that they can be implemented with or without cells. The bundle gap widths were designed to be sufficiently large to allow for adequate cellular infiltration (table 1). Most cells range in size from approximately 10–30 *μ*m in width and up to 200 *μ*m in length [69, 70]. All four of the scaffolds designed in this study have bundle gap widths large enough to allow for cell infiltration. In addition to this, the fibrous scaffolds in this study all allowed for cell infiltration, with both the SEM images (figure 7) and the immunofluorescent images (figure 9) showing evidence of successful NHDF infiltration. While there was variance between the different scaffold groups, infiltration was still noted suggesting that they are all capable of allowing for cell attachment and growth on both sides of the polymer fibers.

The present study showed that our architected polymer scaffolds could be successfully combined with a cell-laden hydrogel, ultimately leading to stable phenotype and cell alignment. The immunofluorescent staining (figure 9) and nuclear alignment analysis (figure 10) showed that the NHDFs encapsulated within the composite scaffold were clearly able to sense the polymer fibers and aligned themselves accordingly. Not only was this noticeable on the top side of the polymer fibers (the side being seeded), but this was also noted on the underside of the polymer fibers. Interestingly, the SEM images of the composite scaffolds not only show cell alignment along the polymer fibers, but also show that the protein constituents (collagen) of the hydrogel aligned themselves along the polymer fibers (figure 10). This phenomenon shows that both the cells (NHDFs in our case) and the hydrogel are affected by the architecture of the polymer fibers that they are encapsulated around. Furthermore, our work looking at co-culturing NHDFs and ECs showed that these composite scaffolds were suitable for the co-culture of different cell-lines on either side of the polymer fibers. Both the NHDFs and ECs showed a phenotypic morphology that would be expected from these cell lines after 7 d of *in vitro* culture [33, 71]. In addition to this, the success of the co-culture system (figure 11) shows that these composite scaffolds can be seeded from both sides separately and still form a solid singular composite scaffold. These results show that our novel architected composite scaffolds are capable of accommodating different cell lines and therefore have potential of use with a range of different tissue types.

Hybrid scaffolds have become more popular in the field of tissue engineering due to their ability to incorporate the benefits of more than one type of scaffold manufacturing technique [72–75]. In this present study, the electrospun scaffold provides stiffness, and due to its novel structure, also provides anisotropy. However, electrospun polymer scaffolds alone often lack the biochemical benefits that other scaffold manufacturing techniques possess. To combat this, studies have shown that hydrogels and electrospun scaffolds can be combined to gain the benefits from both scaffolding techniques [73]. Therefore, the capture device in the present study has been designed so that a cell laden hydrogel can be encapsulated around the electrospun scaffold. However, significant infiltration of the hydrogel with cells would likely lead to proper functional tissue formation as Kaiser *et al* demonstrated using the same encapsulation method as the one presented in this study [72]. In summary, we have developed highly tuneable fibrous PCL scaffolds compatible with cell/hydrogel tissue engineering and have shown through data extrapolation that the anisotropic mechanical properties of mammalian tissues can be recapitulated to most accurately mimic native tissue architecture and mechanics.

## 5. Conclusions

The electrospun PCL scaffolds presented in this study were designed in the form of bundled fibers (bundles containing several fibers) with large gaps between the bundles to ensure cells could infiltrate whilst also maintaining mechanical resistance. Furthermore, the scaffolds demonstrated an array of anisotropic properties with Young’s modulus ratios (Young’s modulus along primary axis vs Young’s modulus along secondary axis) ranging from 1.8 up to 540 when the angle between the two layers of the scaffold changes from 90° to 0°. Through data extrapolation of the scaffolds manufactured in this study, a scaffold can now be designed with the same anisotropic ratio of Young’s moduli to that of any tissue type. In conclusion, these results allow for the anisotropic mechanical properties of all tissue types to be accurately mimicked by electrospinning architected bundled fiber scaffolds.

## Figures and Tables

**Table 1.** Electrospun scaffold properties for the four architected scaffold morphologies.

Target angle (°)	Actual angle (°)	Fiber diameter (*μ*m)	Bundle diameter (*μ*m)	Bundle gap width (*μ*m)
0	0.7 ± 0.4^a^	3.70 ± 0.70	54.9 ± 14.2	51.9 ± 13.3^a^
30	35.8 ± 3.6^a^	3.66 ± 0.61	52.4 ± 19.5	79.6 ± 20.4^a^
60	56.2 ± 1.8^a^	3.44 ± 0.59	35.8 ± 13.2	35.6 ± 12.0
90	88.4 ± 0.8^a^	3.87 ± 0.39	62.5 ± 29.3	36.6 ± 8.6

a Indicates *p* < 0.05 versus all other groups.

**Table 2.** Extrapolated scaffold fiber angles with Young’s moduli that match the anisotropic properties of different tissues found in the literature.

Tissue type	Reference	Tissue processing/analysis method	Anisotropic ratio of Young’s moduli	Extrapolated scaffold fiber angle (°)
Myocardium—rat	[36]	Quasistatic compression on fresh tissue	1.86	82.05
Myocardium—human	[3]	Ultrasonic measurements on formalin fixed tissue	3.25	74.90
Myocardium left ventricular scar—porcine: 12 weeks post myocardial infarction (MI)	[42]	Magnetic Resonance Imagiong (MRI) and Finite Element Analysis (FEA)	2.36	79.00
Myocardium left ventricular scar—porcine: 1 week post MI	[42]	MRI and FEA	7.08	64.93
Liver—porcine	[6]	Quasistatic compression on fresh tissue	1.30	86.64
Articular cartilage (knee)—human	[40]	Quasistatic compression on fresh tissue	1.47	85.06
Kidney—human	[43]	Quasistatic tension on fresh tissue	1.89	81.85
Skin—human lower back	[44]	Quasistatic tension on fresh tissue	1.96	81.38
Skeletal muscle—goat	[45]	Quasistatic tension on fresh tissue and FEA	3.70	73.24
Saphenous vein—human	[46]	Quasistatic tension on fresh tissue	5.64	67.84
Aortic heart valve—human	[41]	Quasistatic tension on fresh tissue	7.80	63.67
Aortic heart valve—human	[47]	Quasistatic tension on fresh tissue	9.27	61.47
Tendon—human	[3]	Ultrasonic measurements on formalin fixed tissue	19.41	52.00

## Data Availability

The data that support the findings of this study are available upon reasonable request from the authors.
